# Reply: Evaluation of management of desmoid tumours associated with familial adenomatous polyposis in Dutch patients

**DOI:** 10.1038/bjc.2011.51

**Published:** 2011-03-01

**Authors:** M H Nieuwenhuis, H F A Vasen

**Affiliations:** 1 The Netherlands Foundation for the Detection of Hereditary Tumours, Rijnsburgerweg 10, Poortgebouw Zuid, AA Leiden 2333, The Netherlands; 2 Department of Gastroenterology and Hepatology, Leiden University, Medical Centre, Albinusdreef 2, ZA Leiden 2333, The Netherlands

Sir,

We thank Bhandari *et al* (2011) for their valuable comments on our paper (Nieuwenhuis *et al*, 2011). In this study, we evaluated the effectiveness of treatment in 78 patients, known at the Dutch Polyposis Registry, with desmoids diagnosed between 1978 and 2010. The results indicated that for intra-abdominal desmoid tumours, both a conservative approach and surgery led to comparable outcomes, and that also the effect of various pharmacological therapies was similar.

The first question of Bhandari *et al* (2011) addressed the indications of surgery, whether a stepwise approach was used and whether the patients received medical treatment before surgery. The indications for surgery were severe clinical symptoms or the large size of the desmoid tumour. Owing to the retrospective nature of the study, it is possible that the indications for the surgery group and the medical treatment group may slightly differ. However, characteristics of both groups (sex, age at diagnosis, and median size of the desmoids) were similar. The stepwise approach, that is, starting with medical treatment followed by surgery in case of no response, was not used in most cases as this approach was only recently recommended (Sturt and Clark, 2006; Vasen *et al*, 2008) and our study included patients that were diagnosed over a period of >20 years. Another reason was that a considerable proportion of patients were treated in local hospitals, making standardised treatment less likely. None of the patients who underwent surgery had previous history of medical treatment. Patients who had received medical therapy before surgery were classified in the ‘non-surgery’ group.

Another question was which type of surgery was performed. In all cases, the intention was to remove the mesentery desmoid tumour. If this was impossible, then only a bypass was carried out in case of obstruction. Unfortunately, in none of the patients it was possible to remove all desmoid tumour tissue.

The final question was about the definition of a progression-free survival (PFS). In our study, PFS was defined as the period between the start of medical treatment or the time of surgery and the beginning of clinical symptoms attributed to growth of the desmoid. We realise that this is not a standardised method for measuring tumour progression. However, we think that the presence of new complaints because of tumour growth is a clinically important parameter. In many cases, tumour growth was confirmed by MRI or by CT scanning. However, we agree with Bhandari *et al* (2011) that in familial adenomatous polyposis patients, it is sometimes difficult to attribute abdominal complaints to the desmoid tumour, because most patients had also colorectal surgery and some of them may have complaints associated with adhesions.

We very much appreciate the critical notes of Bhandari *et al* (2011) and fully realise that most questions can only be completely addressed by performing a prospective study.
